# Investigation of 3D-Moldability of Flax Fiber Reinforced Beech Plywood

**DOI:** 10.3390/polym12122852

**Published:** 2020-11-29

**Authors:** Johannes Jorda, Günther Kain, Marius-Catalin Barbu, Matthias Haupt, Ľuboš Krišťák

**Affiliations:** 1 Forest Products Technology and Timber Construction Department, Salzburg University of Applied Sciences, Markt 136a, 5431 Kuchl, Austria; jjorda.lba@fh-salzburg.ac.at (J.J.); marius.barbu@fh-salzburg.ac.at (M.-C.B.); mhaupt.htw-m2016@fh-salzburg.ac.at (M.H.); 2 Department of Wood Science and Technology, Mendel University, Zemědělská 3, 61300 Brno, Czech Republic; 3 Faculty for Furniture Design and Wood Engineering, Transilvania University of Brasov, B-dul. Eroilor nr. 29, 500036 Brasov, Romania; 4 Faculty of Wood Sciences and Technology, Technical University in Zvolen, T. G. Masaryka 24, SK-960 01 Zvolen, Slovakia; kristak@tuzvo.sk

**Keywords:** plywood, veneer 3D moldability, natural fiber reinforcement

## Abstract

The current work deals with three dimensionally molded plywood formed parts. These are prepared in two different geometries using cut-outs and relief cuts in the areas of the highest deformation. Moreover, the effect of flax fiber reinforcement on the occurrence and position of cracks, delamination, maximum load capacity, and on the modulus of elasticity is studied. The results show that designs with cut-outs are to be preferred when molding complex geometries and that flax fiber reinforcement is a promising way of increasing load capacity and stiffness of plywood formed parts by respectively 76 and 38% on average.

## 1. Introduction

The world is not flat-driven by the rising consumer awareness for the ecological product footprint, designers are set to bid and overcome the limits for material applications. Composite materials are developed to surpass single inferior material properties in order to combine quality characteristics for specific maximum performance. A broadly available, natural composite material resource is wood-defined as a natural polymeric, cellular fiber composite with superior advantages compared to other engineering materials [1]. Various wood-based products, such as cross laminated timber (CLT), particleboard, oriented strand boards (OSB) or fiberboards, resolve solid wood disadvantages, respectively anisotropy, biodegradability, and dimensional limitations [2].

Plywood, with its multilayered veneer-based laminar structure, is considered to be the oldest and the most important wood-based composite material with two distinct fields of application—construction purposes and multidimensional forming for interior and exterior products. Plywood can be bent by steaming a panel before forming, thin panels can be threaded and glued together or veneer layers are mechanically modified when producing 3D-molded parts [3]. To enhance veneer-based products (plywood and laminated veneer lumber) mechanical load bearing capacity, several experimental studies were conducted addressing synthetic glass, carbon, or other artificial fiber reinforcement [4,5,6,7,8]. Results revealed significantly improved mechanical properties for modulus of elasticity (MOE) and modulus of rupture (MOR), dimensional stability, and especially splitting strength [9,10,11]. Currently, the focus on fiber reinforcement shifted to more ecofriendly, fiber sources based on renewable resources. Flax (*Linum usitatissium*), ramie (*Boehmeria nivea*), hemp (*Cannabis sativa*), sisal (*Agave sisalana*), kenaf (*Hisbiscus cannabinus*), jute (*Corhorus capsularis*) or bamboo are characterized by biodegradability, cost effectiveness, natural availability [12,13]. Natural fibers are emerging at low cost, lightweight and apparently environmentally superior alternatives to glass fibers in composites. The current mass-based price of raw processed glass fibers with a density of 2.6 g/cm^3^ is approximately 2 to 4 times greater than that of e.g., flax fibers with a density of 1.5 g/cm^3^ [14,15]. The elevated mechanical properties of lightness, stiffness to weight ratio, and high tensile strength of natural fibers are advantageous for high performance composites [16,17]. For instance, flax with a density of 1.50 g/cm^3^, tensile strength between 345 to 1100 N/mm^2^, and an MOE of 27,600 to 64,500 N/mm^2^ [18] is characterized by a relatively high stiffness, load capacity, and damping performance and shows comparable mechanical performance as e-glass and s-glass fibers. Moreover, flax is flexible and preferred for complicated geometries [19]. Bőhm et al. [20] evaluated the bending characteristics of sandwich composite materials based on balsa plywood reinforced with flax (2 × 2 twill, 400 g/m^2^) and glass fibers (roving fabric, 390 g/m^2^) with epoxy resin. Reinforcement of the balsa plywood with both types of fibers significantly increased the modulus of elasticity and bending strength. Whilst for flax fibers, the flexural modulus was 7.5% higher than for glass fibers, the bending strength values of glass fibers were 26.5% lower compared to flax fibers. Research confirmed that flax fibers with less environmental impact may be a material that is used as an adequate replacement of glass fibers. Papadopoulos and Hague [21] prepared single-layer particleboards made from various wood chip/flax mixtures bonded with urea formaldehyde resin. The strength properties of boards containing up to 30% (mass-based) flax particles meet the minimum European standard (EN 310, 317, 319) requirements for interior particleboards. In the research of Susainathan et al. [22,23], glass fiber, carbon fiber, and flax fiber were used as the surface layer, poplar and okoume as the core layer to make wood-based sandwich structures. The impact and bending properties of the boards with flax fiber reinforcement showed comparable bending strength and stiffness as glass fiber reinforced samples. Research on fiber reinforcement primarily focuses on two-dimensional panel applications [24,25,26].

Bendability (formability) of two-dimensional curvature for wood is well investigated [27]. Three-dimensional (3D) molding of wood is considered as one of the most complicated chipless methods of shaping wood [28,29]. In general, limitations for veneer formability are set by the anisotropic character of wood and influenced by the MOE and tensile strength for the two directions parallel and perpendicular to grain. Wagenführ et al. [30] revealed a correlation between bending radius and veneer thickness for two-dimensional bending with a significant increase for the minimal possible radius with increasing veneer thickness and dependence on grain direction for non-modified veneers. Additional research on non-modified two-dimensional veneer bending suggested a higher suitability of tangential veneers for molding [31]. 3D veneer moldability is furthermore limited by the anisotropic nature, varying tensile strength, and plastic deformation of veneers [30]. Other relevant parameters are wood species, moisture content, sample and molding geometry in this respect. Deciduous wood species like beech (*Fagus sylvatica* L.) are considered more suitable for 3D-molding due to wood anatomy and plasticizing ability. Wood moisture content, heat pressing, moistening of wood are process variables which are relevant for surface quality in 3D wood molding [32]. It was shown that the 3D formability of veneer can be improved by 12 to 50% by increasing veneer moisture content and steaming. Birch (*Betula pendula Roth*) veneer showed better forming properties than beech (*Fagus sylvatica* L.) and ash (*Fraxinus excelsior*) [33]. Warping and cracking in the forming process depends strongly on veneer thickness [29]. According to Fekiač and Gáborík [27], there is no clear preference between radial and tangential veneers for 3D molding. Another way to increase the moldability of the wood is surface densification, hydrothermal plasticizing, thermal modification, reinforcement by natural fibers, and the optimization of the pressing process [34]. Zerbst et al. [35] successfully applied the Nakjima test to evaluate the deep drawing capacity of veneer laminate, which can be used for forming simulations. The authors found that cracks in veneer laminate sheets occur in the early wood zone and propagate in grain direction.

The aim of the study is to determine the influence of woven natural flax fiber reinforcement on 3D molded beech veneer-based plywood with a specific molding geometry. The occurrence of surface cracks and delamination as well as 3D-bending behavior for maximum load and MOE with the factors mold geometry and fiber-reinforcement are investigated.

## 2. Materials and Methods

### 2.1. Sample Preparation

**Figure 1:**
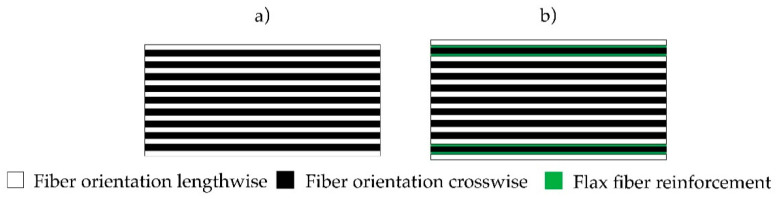
Veneer layup reference (**a**), with flax fiber reinforcement (**b**).

Pre-conditioned (20 °C, 65% relative humidity) radial sliced zero defect beech (*Fagus sylvatica* L.) veneers with a thickness of 0.6 mm, an average density of 0.67 g/cm^3^, and an average moisture content of 10.2 (SD = 0.3) % were used as wooden raw material in this study due to its superior bending strength and MOE performance compared to birch (*Betula pendula Roth)*. Twill flax fabric LINEO FlaxPly Balanced Fabric 200 (Ecotechnilin, Valliquerville, France) with a thickness of 0.4 mm, a density of 1.27 g/cm^3^, and a grammage of 200 g/m^2^ acted as fiber reinforcement. The flax moisture content accounted for 11 (SD = 0.3) %. West Systems International (Romsey, England) 105 Epoxy Resin and 207 Special Coating Hardener were used as adhesive. Two kinds of lay-ups were introduced (Figure 1). The unreinforced reference samples existed of ninety degree cross layered veneer layers, whereas the flax fiber reinforced samples consisted of the identical ninety cross layered veneer layers with additional four layers of flax fabric. These were located at the first and second glue line on each side in order to improve the tensile strength under bending and to minimize the influence of shear stresses. The calculated amount of epoxy resin per glue line was set to 200 g/m² for the veneer to veneer layers and 400 g/m^2^ for flax fiber to veneer.

**Figure 2:**
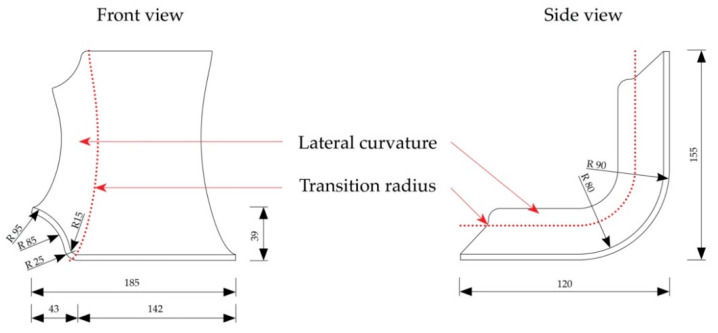
Dimension of the 3D molded specimens (dimensions in mm).

The specimens were designed focusing on the use of the molded parts as car seat shells (Figure 2). A transition section between seating and back was considered in the design of the specimens. The seating shell was split in the center, and looking in driving direction, the left part of the shell was considered. The size of the raw veneer sheets accounted for 20 × 27.5 cm.

**Figure 3:**
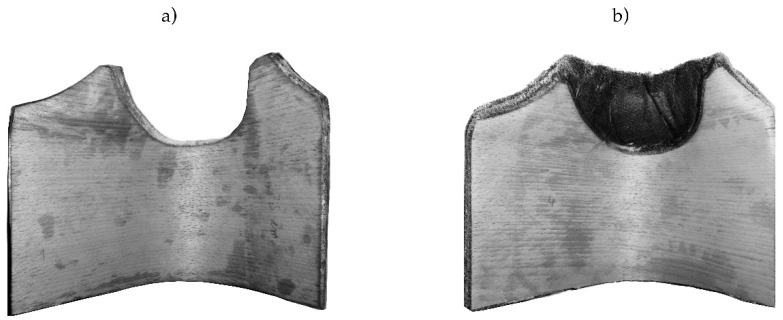
Cut-out of specimens (**a**) reference, (**b**) with flax fiber reinforcement.

To overcome the veneer warping during molding, two different options where used. First, a minimal leaving out of the critical curvature was applied (Figure 3).

**Figure 4:**
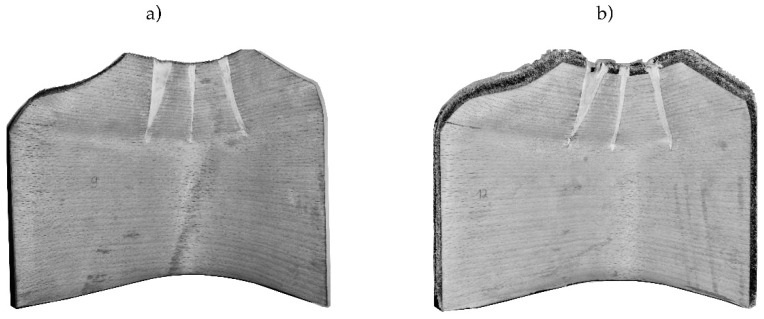
Relief cut of specimens (**a**) reference, (**b**) with flax fiber reinforcement.

Second, relief cuts at the critical curvature were provided (Figure 4). These were filled with an Epoxy filler (Molto 2-components wood replacement) after pressing. In total, four different types of setups with three samples each were tested.

The lay-up of the veneer laminates and the glue application were conducted manually. The calculated areal glue amount spreading per layer was checked by a scales KERN PRS 620-6 (Kern & Sohn GmbH, Ballingen-Frommern, Germany). The controlled molding was carried out using a HÖFLER (Taiskirchen, Austria) HLOP 280 press to reach the target thickness of 10 mm within 15 h of cold pressing. The applied pressure during molding was 2.5 N/mm^2^ for the reference samples and 2.8 N/mm^2^ for the flax fiber reinforced samples. The temperature was kept constant at 20 °C. No pretreatment of the veneers was applied to improve the bending performance. Before further testing, the samples were stored for seven days under constant standard climate conditions (20 °C, 65% relative humidity).

### 2.2. Testing of Surface Cracks and Delamination

Surface cracks and delamination were determined after pressing and conditioning of the formed parts. Samples were trimmed to equal size before proceeding to the 3D bending test.

To determine the surface crack length and widths, pictures were taken with a digital camera (Rollei Compactline 750) and a scale positioned on the formed parts. For the exact determination of length and width, MATLAB R2018a was used according to Equation (1). On each picture taken, a scale was installed, to determine the dimensions of cracks and delamination considering the pixel-based length of the defect $l_{pixel}$, the pixel-based length of the reference scale $l_{ref.\;pixel}$, and the known length of the scale $l_{ref.}$ The delamination length was determined using the identical formula. In addition, a verbal nomenklatura was introduced to describe the location of surface cracks and delamination in order to locate critical spots of failure. First of all, cracks and delamination were categorized whether they occurred (1–1) on the front or (1–2) back of the formed part. Second, it was described whether they occurred (2–1) on the back shell or (2–2) on the seat. Third, defects were assessed regarding their position relative to the transition radius; (3–1) transition radius, (3–2) above transition radius, (3–3) below transition radius. Finally, defects on edges were assessed using the attributes (4–1) outer edge, (4–2) edge cut-out. All measurements were taken from formed parts to exclude influences caused by post-processing.

(1) $$l=\frac{l_{pixel}}{l_{ref.\;pixel}}\times l_{ref.}$$

### 2.3. Three-Dimensional Bending Test

**Figure 5:**
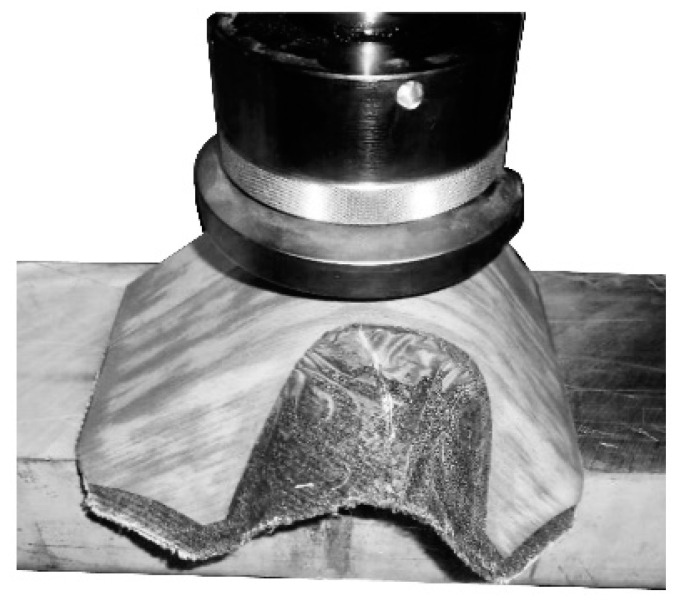
Bending test for 3D molded plywood samples.

A modified bending test following UN/ECE Regulations Nr. 17 [36] with a variation regarding the direction of force for spreading was implemented. For testing, the samples were placed with the small faces on an aluminum block due to its low friction coefficient to reduce influences on internal stress distribution (Figure 5). Constant linear force with a speed of 10 mm/min was applied by a pressure disc (ø 135 mm) parallel to the supports using a Zwick/Röll Z 250. The maximum load (F_max_) and the modulus of elasticity (MOE) were determined following EN 310:2005 [37].

The statistical significance of the factors molding geometry and fiber-reinforcement was determined using two-way ANOVAs with consideration of interaction effects of first order.

## 3. Results and Discussion

### 3.1. Number of Surface Cracks and Delamination

The finished specimens showed up to 6 cracks and between 1 and 2 delamination after pressing. Comparing the number of counted surface cracks for each of the four groups, it was assumed that there is an influence of the factors molding geometry and fiber reinforcement. Based on a two-way ANOVA to prove the statistical significance of the factors molding geometry (*p*-value 0.074) and fiber reinforcement (*p*-value 0.334) displayed that there is no significant influence. In addition, there is no significant interaction effect of the factors. This can be explained by the high variation of crack numbers between specimens (Table 1).

To determine the influence of the factors molding geometry and fiber reinforcement on the number of delamination, the same procedure as for the surface cracks was applied. The two-way ANOVA displayed a significant influence of the molding geometry with a *p*-value of 0.037. On average, formed parts with a cut-out showed one delamination, whilst parts with relief cuts had two delaminations after pressing (Table 2). The fiber-reinforcement (*p*-value 0.631) such as the factor interaction (*p*-value 0.631) in contrast had no significant influence on the number of delaminations.

Considering these statistics, it seems that the cracks and delamination are predominantly caused by deformations occurring during the molding process rather than ineffective gluing between composite layers.

### 3.2. Length and Width of Surface Cracks and Delamination

The effect of the factors molding geometry and fiber reinforcement on the length of cracks was assessed using a two-way ANOVA. Only specimens with cracks were considered. According to the ANOVA, the *p*-value for the interaction between the factors is significant with 0.030, stating the fact that the level effect of one factor is dependent on the level of the other factor. Cracks on fiber reinforced parts with cut-out have an average length of 6.7 (SD = 2.3) mm, 64% lower than their unreinforced counterpart—a coherence that could not be shown for formed parts with relief cuts.

In contrast, the results of the surface crack widths had no statistically significant factor effect or interaction (*p*-value 0.196) between the two factors molding geometry and fiber reinforcement. This is underlined by the singular *p*-value for molding geometry with 0.774 and fiber reinforcement with a *p*-value 0.864.

Based on the comparison of the mean and standard deviations of the factors, the two-way ANOVA revealed no statistically significant influence on the delamination length. Molding geometry (*p*-value 0.587) and fiber reinforcement (*p*-value 0.203) showed no significant interaction effect (*p*-value of 0.450).

The sum of the crack length of a specimen is significantly (*p*-value 0.034) affected by the molding geometry, showing that it accounts for 16.6 (SD = 27.9) mm with formed parts with cut-out compared to 64.6 (SD = 33.5) mm with relief cut. The cumulated crack width per sample is not significantly affected by the mold geometry or fiber reinforcement. The effect of molding geometry on the cumulated length of delamination is narrowly not significant (*p*-value 0.053). The average sum of delamination length accounts for 12.9 (SD = 10.5) mm when using cut-outs and 23.6 (SD = 4.0) mm when relief cuts are applied (Table 3). Summarized, it was found that molded parts with relief cut have more and additionally more significant cracks and delamination than parts with a cut-out. The sum of the crack lengths is two thirds lower when applying fiber reinforcement for parts with cut-out, whereas no advantage of fiber reinforcement could be detected for parts with relief cuts. The investigation of cracks and delamination shows that the total sum of defect characteristics is higher for parts with relief cuts. This is due to the fact that a greater part of the critical sector of the transition zone is covered. The forced deformations are greater with the relief cut formed parts and exceed more often the strength of the veneer. The fiber reinforced parts with cut-out show lower crack and delamination length, probably due to the fact that fabric with twill weave is easily shear formable and has good draping properties [38].

### 3.3. Location and Orientation of Defects

**Figure 6:**
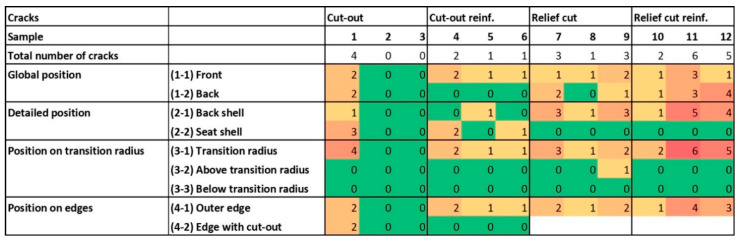
Heat map of crack number and position.

Surface cracks occurred with a relative frequency of 67 (SD = 31) % on the inner (positive) side of the molding with the shorter radius. Ninety-seven (SD = 11) percent are located at the “transition radius” due to the sharp radius and the change of direction into three different dimensions. Seventy-six (SD = 22) percent are placed on the outer edges of the molding (Figure 6). The reason is, that tensions cannot be transferred to neighboring veneer layers in the corner range which results in cracks. All surface cracks are oriented in grain direction, because the tensile strength of beech veneer orthogonally to grain direction is 20 times lower than in grain direction [39], which is confirmed by Zerbst et al. [35] finding the same phenomenon when producing molded veneer laminates.

Cracks are not acceptable in molded parts due to structural and aesthetic reasons. A possible optimization is to create micro cracks and collapsed cells in veneer layers before molding to prevent the formation of macro cracks. It is important to note that a collapsed cell not necessarily goes along with a broken cell wall [30].

**Figure 7:**
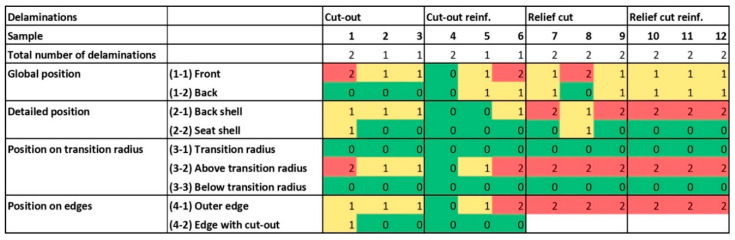
Heat map of delamination number and position in the 3D-molded plywood samples after forming.

Delaminations were predominantly found on the inner (front) side as well (Figure 7). Seventy-seven (SD = 26) percent occurred on the more strongly bent inner side. All of them were found above the transition radius on the formed parts where the shear strengths were strongest. For the same reason, these occurred in large part with some distance to the cut-out referenced to the edge position. Ninety-five percent of all delaminations were found near the outer edge of the formed parts. The use of rigid press forms results in an uneven pressure distribution because the application of uniaxial vertical pressure results in uneven orthogonal pressure referred to the curved surface of the formed part [40]. The maximal angle of the form parts used in this study is 50 degrees on the outer edge and the pressure was therefore 36% [40] reduced in this area, as an explanation for the less efficient bonding. The strong influence of the mold geometry on the quality of plywood formed parts was discussed by Comsa [41], suggesting deformation modeling using finite element methods. This shows an interesting opportunity for the current mold part to lower crack and delamination formation during molding. Finally, the use of plasticized veneer (at least the outer layers) before pressing suggests to lower the risk for cracks [34].

### 3.4. 3D Bending Test

**Figure 8:**
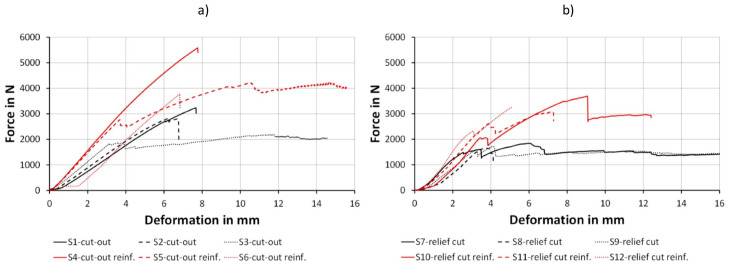
Force-path diagram for specimens with cut-out (**a**) and relief cut (**b**).

Based on the load-deformation curvature, two thirds of the samples with cut-outs showed a linear-elastic deformation behavior and a nondescriptive fracture with a mixture of delaminations and failures within the beech veneer (Figure 8). One third of samples had a partial fracture behavior within the linear-elastic deformation with a notable tension failure. The load-deformation curve for relief cut samples revealed a semi-fracture at the end of the linear-elastic range caused by the failure of the filler regardless of fiber reinforcement, followed by a further plastic deformation. Fiber reinforcement resulted in an increase in force for both cut-out and relief cut samples in the plastic deformation range, whereas unreinforced samples of both groups more or less stagnated. These findings validate research by Wagenführ et al. [30] considering that veneer laminates behave predominantly elastically.

The results of maximum load displayed a significant influence of the molding geometry (*p*-value 0.010) as well as the fiber reinforcement (*p*-value 0.001). Moldings with cut-outs resulted in 58 and 36% higher bending strength than their counterparts with relief cuts. Fiber reinforcement lead to 65 and 92% higher bending strength compared with the unreinforced part, respectively (Table 4).

The reason for the significant increase in the maximal load capacity through flax fiber reinforcement is the high tensile strength of flax fibers, approximately 5 times as high as the one of the wood fiber [38]. This increased tensile strength affects especially the inner side of the formed part which is tensile-loaded in the current experimental situation. As the relief cuts are an interruption in the fiber reinforcement, the filler grouts fail early and are an explanation for the lower performance of such parts.

Regarding the results of the MOE, similar effects are given. The influence of the fiber reinforcement is highly significant (*p*-value 0.001), whereas the influence of the molding geometry—in contrast to the maximum load capacity—was statistically not significant (*p*-value 0.168). Formed parts which are fiber reinforced had an MOE on average 76% higher than the unreinforced parts (Table 4). The mold geometry does not have an effect in this respect, because failures occur above 40% of the maximal force and up to this point the part shows linear-elastic behavior.

A significant correlation between the sum of crack width in a part and the MOE was observed (*p*-value 0.024, R^2^ = 0.41). The higher the cumulated crack width, the higher the MOE. Moreover, a higher number of cracks result in a not significant higher MOE. A rationale for this coherence is that crack formation during the forming process results in an improved layer contact due to a compensation of the deformations in the transition zones.

## 4. Conclusions

The three-dimensional bending tests regarding the maximum load capacity and the MOE showed that the performance of beech veneer-based 3D molded plywood could be significantly improved by flax fiber reinforcement. In addition, the influence of the mold geometry is significant for the load capacity, but not for the MOE.

Formed parts with few and less pronounced defects, such as cracks and delamination, can be produced using cut-outs in the most deformed area of the 3D molded parts. The location of cracks and delamination found in this study strongly suggests the optimization of the press form and the application of multiaxial press processes to avoid delamination. Moreover, 3D molded parts should be produced with some oversize as 68% of the cracks and 95% of the delamination was found on the outer edges. A majority of defects could be removed in a final trimming process.

Further research is recommended for a deeper understanding of the interaction and influence of veneer thickness and flax fabrics grammage on formability, as well as the influence of veneer pretreatment and hot pressing to improve 3D formability. Additional research should focus on interactions between veneer, flax, and adhesive to determine a better understanding of delamination resistance and supplemented by an evaluation of fiber wetting resulting from the resin. Cost and production aspects have to be taken into account for broader industrial applications. Especially the research on cost efficient industrial glue systems, glue amount optimization, and the influence on the performance of woven flax fiber fabric is worth further research.

## Figures and Tables

**Table 1:** Number of cracks and crack geometry after bending (SD in brackets).

Specimen	No. of Cracks	Crack Length	Crack Width	N
		(mm)	(mm)	
Cut-out	1 (2)	18.2 (7.0)	0.6 (0.4)	3
Cut-out reinf.	1 (1)	6.7 (2.3)	0.4 (0.2)	3
Relief cut	2 (1)	12.2 (3.9)	0.4 (0.1)	3
Relief cut reinf.	4 (2)	17.4 (11.1)	0.5 (0.3)	3

**Table 2:** Number and length of delamination after bending (SD in brackets).

Specimen	No. of Delamination	Delamination Length	N
		(mm)	
Cut-out	1 (1)	12.7 (4.8)	3
Cut-out reinf.	1 (1)	8.9 (3.4)	3
Relief cut	2 (0)	12.3 (3.3)	3
Relief cut reinf.	2 (0)	11.3 (3.4)	3

**Table 3:** Number and length of delamination after bending (SD in brackets).

Specimen	Sum Crack Length	Sum Crack Width	Sum Delam. Length	N
	(mm)	(mm)	(mm)	
Cut-out	24.2 (41.9)	0.6 (1.0)	16.9 (13.0)	3
Cut-out reinf.	8.9 (4.1)	1.1 (0.9)	8.9 (7.8)	3
Relief cut	53.9 (38.5)	0.6 (0.4)	24.6 (5.4)	3
Relief cut reinf.	75.3 (31.3)	2.2 (1.4)	22.6 (2.8)	3

**Table 4:** Results of the bending test (SD in brackets).

Specimen	Max. Force	MOE	N
	(N)	(N/mm^2^)	
Cut-out	2784 (530)	532 (45)	3
Cut-out reinf.	4531 (941)	791 (68)	3
Relief cut	1736 (97)	633 (70)	3
Relief cut reinf.	3340 (322)	816 (97)	3

## References

[1] Mahut J., Reh R. (2007). Plywood and Decorative Veneers.

[2] Stark N.M., Cai Z., Carll C. (2010). Chapter 11—Wood-Based-Composite Materials and Panel Products, Glued Laminated Timber, Structural Materials. Wood Handbook—Wood as an Engineering Material.

[3] Panic L., Hodzic A., Nezirevic E. (2016). Modern and sophisticated processes of 3D veneer plywood bending. Acta Tech. Corviniensis Bull. Eng..

[4] Muthuraj R., Misra M., Defersha F.M., Mohanty A.K. (2016). Influence of processing parameters on the impact strength of biocomposites: A statistical approach. Compos. Part A Appl. Sci. Manuf..

[5] Percin O., Altunok M. (2017). Some physical and mechanical properties of laminated veneer lumber reinforced with carbon fiber using heat-treated beech veneer. Holz Roh Werkst..

[6] Liu H., Luo B., Shen S., Liu H. (2018). Design and mechanical tests of basalt fiber cloth with MAH grafted reinforced bamboo and poplar veneer composite. Holz Roh Werkst..

[7] Auriga R., Gumowska A., Szymanowski K., Wronka A., Robles E., Ocipka P., Kowaluk G. (2020). Performance properties of plywood composites reinforced with carbon fibers. Compos. Struct..

[8] Liu Y., Guan M., Chen X., Zhang Y., Zhou M. (2019). Flexural properties evaluation of carbon-fiber fabric reinforced poplar/eucalyptus composite plywood formwork. Compos. Struct..

[9] Xu H., Nakao T., Tanaka C., Yoshinobu M., Katayama H. (1998). Effects of fiber length and orientation on elasticity of fiber-reinforced plywood. J. Wood Sci..

[10] Rowlands R.E., Deweghe R.P., Laufenberg T.L., Krueger G.P. (1986). Fiber-reinforced wood composites. Wood Fiber Sci..

[11] Bal B.C., Bektaş I., Mengeloğlu F., Karakuş K., Demir H.Ö. (2015). Some technological properties of poplar plywood panels reinforced with glass fiber fabric. Constr. Build. Mater..

[12] Sorieul M., Dickson A.R., Hill S.J., Pearson H. (2016). Plant Fibre: Molecular Structure and Biomechanical Properties, of a Complex Living Material, Influencing Its Deconstruction towards a Biobased Composite. Materials.

[13] Ticoalu A., Aravinthan T., Cardona F. A Reviewof Current Development in Natural Fiber A Review of Current Development in Natural Fiber Composites for Structural and Infrastructure Applications. Proceedings of the Southern Region Engineering Conference.

[14] Šedivka P., Bomba J., Böhm M., Zeidler A. (2015). Determination of Strength Characteristics of Construction Timber Strengthened with Carbon and Glass Fibre Composite Using a Destructive Method. Bioresources.

[15] Joshi S., Drzal L., Mohanty A., Arora S. (2004). Are natural fiber composites environmentally superior to glass fiber reinforced composites?. Compos. Part A Appl. Sci. Manuf..

[16] Borri A., Corradi M., Speranzini E. (2013). Reinforcement of wood with natural fibers. Compos. Part B Eng..

[17] Sam-Brew S., Smith G. (2015). Flax and Hemp fiber-reinforced particleboard. Ind. Crops Prod..

[18] Mohanty A.K., Misra M., Hinrichsen G. (2000). Biofibres, biodegradable polymers and biocomposites: An overview. Macromol. Mater. Eng..

[19] Goudenhooft C., Bourmaud A., Baley C. (2019). Flax (*Linum usitatissimum* L.) Fibers for Composite Reinforcement: Exploring the Link between Plant Growth, Cell Walls Development, and Fiber Properties. Front. Plant Sci..

[20] Böhm M., Brejcha V., Jerman M., Černý R. Bending Characteristics of Fiber-Reinforced Composite with Plywood Balsa Core. Proceedings of the International Conference of Computational Methods in Sciences and Engineering 2019 (ICCMSE-2019).

[21] Papadopoulos A.N., Hague J.R. (2003). The potential for using flax (*Linum usitatissimum* L.) shiv as a lignocellulosic raw material for particleboard. Ind. Crops Prod..

[22] Susainathan J., Eyma F., De Luycker E., Cantarel A., Castanié B. (2018). Experimental investigation of impact behavior of wood-based sandwich structures. Compos. Part A Appl. Sci. Manuf..

[23] Susainathan J., Eyma F., De Luycker E., Cantarel A., Castanier B. (2017). Manufacturing and quasi-static bending behavior of wood-based sandwich structures. Compos. Struct..

[24] Mathijsen D. (2018). The renaissance of flax fibers. Reinf. Plast..

[25] Prabhakaran S., Krishnaraj V., Sharma S., Senthilkumar M., Jegathishkumar R., Zitoune R. (2019). Experimental study on thermal and morphological analyses of green composite sandwich made of flax and agglomerated cork. J. Therm. Anal. Calorim..

[26] Jorda J.S., Barbu M.C., Kral P. (2019). Natural fiber reinforced veneer based products. Pro Ligno.

[27] Fekiac J., Gáborík J. (2016). Formability of Radial and Tangential Beech Veneers.

[28] Wagenführ A., Buchelt B. (2005). Untersuchungen zum Materialverhalten beim dreidimensionalen Formen von Furnier. Holztechnologie.

[29] Gaff M., Gašparík M. (2017). 3D Molding of Veneers by Mechanical and Pneumatic Methods. Materials.

[30] Wagenführ A., Buchelt B., Pfriem A. (2005). Material behaviour of veneer during multidimensional moulding. Holz Roh Werkst..

[31] Langova N., Joscak P., Mozuchova M., Trencanova L. (2013). Analysis the effects of bending load of veneers for purposes of planar moulding. Ann. Wars. Univ. Life Sci..

[32] Gaff M., Gáborík J. (2014). Evaluation of Wood Surface Quality after 3D Molding of Wood by Pressing. Bioresources.

[33] Fekiac J., Gáborík J., Smidriakova M. (2016). 3D formability of moistened and steamed veneers. Acta Fac. Xylologiae Zvolen.

[34] Zemiar J., Fekiac J., Gaborik J., Petro A. (2013). Three-dimensional formability of rolled, pressed, and plasticized veneers. Ann. Wars. Univ. Life Sci..

[35] Zerbst D., Affronti E., Gereke T., Buchelt B., Clauß S., Merklein M., Cherif C. (2020). Experimental analysis of the forming behavior of ash wood veneer with nonwoven backings. Holz Roh Werkst..

[36] United Nations Economic Commission for Europe Regulation No 17 of the Economic Commission for Europe of the United Nations (UN/ECE)—Uniform Provisions Concerning the Approval of Vehicles with Regard to the Seats, Their Anchorages and Any Head Restraints. https://op.europa.eu/en/publication-detail/-/publication/4d5ab93c-7d45-4b3a-b49f-b10b6476b5df.

[37] European Committee for Standardization (2005). EN 310:2005 Wood Based Panels—Determination of Modulus of Elasticity in Bending and of Bending Strength.

[38] Schürmann H. (2007). Konstruieren Mit Faser-Kunststoff-Verbunden.

[39] Wagenführ R. (2006). Holzatlas.

[40] Kollmann F. (1955). Technologie des Holzes und der Holzwerkstoffe.

[41] Comsa G.N. Dimensional and geometrical optimization of structures and materials for curved or molded chair furniture. Proceedings of the 3rd International Conference on Advanced Composite Materials Engineering COMAT.

